# Stigma Resistance and Its Associated Factors among Patients with Mood Disorder at St. Paul's Hospital and Millennium Medical College, Addis Ababa, Ethiopia, 2019

**DOI:** 10.1155/2020/7429567

**Published:** 2020-06-09

**Authors:** Elias Tesfaye, Chalachew Kassaw, Liyew Agenagnew

**Affiliations:** ^1^Department of Psychiatry, Faculty of Medical Sciences, Institute of Health, Jimma University, Jimma, Ethiopia; ^2^Department of Psychiatry, College of Health Science, Dilla University, Dilla, Ethiopia

## Abstract

**Background:**

Stigma resistance is described as the capacity to counteract or remain unaffected by the stigma of mental illness. Patients who have high stigma resistance have shown good treatment outcome, so working on this issue is crucial since little is known about the stigma resistance level among patients with mood disorders.

**Objectives:**

To determine the magnitude and determinant factors of stigma resistance among patients with mood disorder attending at St. Paul's Hospital.

**Methods:**

A cross-sectional study design was conducted on 238 study samples, and systematic random sampling was used to get the study participants. Internalized Stigma of Mental Illness Scale was used to measure stigma resistance. Data was entered using EpiData 3.1 and exported to the Statistical Package for Social Science 22.0 for analysis. Linear regression analysis (*P* < 0.05) was used to identify a significant association between the outcome and predictor variable.

**Results:**

Out of 238 study samples, 235 patients took part with a 99% response rate. The overall percentage of stigma resistance was 49.5%. Low educational status (*B* = −1.465, 95% CI (-2.796, -0.134), *P* ≤ 0.031), disability (*B* = −0.064, 95% CI (-0.102, -0.026), *P* ≤ 0.001), nonadherence due to stigma (*B* = −1.365, 95% CI (-2.151, -0.580), *P* ≤ 0.001), duration of treatment (*B* = 0.091, 95% CI (0.042, 0.141), *P* ≤ 0.001), internalized stigma (*B* = −2.948, 95% CI (-3.642, -2.254), *P* ≤ 0.001), and self-esteem (*B* = 1.859, 95% CI (0.812, 2.906), *P* ≤ 0.001) were significantly associated with stigma resistance.

**Conclusion:**

This study found that only half of the patients had stigma resistance. Low educational status, high self-stigma, low self-esteem, disability, and short duration of treatment were negatively associated with stigma resistance, so working on those modifiable identified factors with focal stakeholders will be crucial to promote the stigma resistance level of patients with mood disorder.

## 1. Introduction

According to a 2019 World Health Organization report, the current prevalence of mental illness in the adult population is 22.1%, and from this, more than half accounts for mood disorder (bipolar and depression) which is a recurrent chronic disorder characterized by fluctuations in mood state and energy [1].

Stigma is defined as a mark of shame, disgrace, or disapproval which results in an individual being rejected, discriminated against, and excluded from participating in a number of different areas of society [2]. Around the world, negative attitudes, beliefs, and behaviors are endorsed in patients with all groups of mental illness, and some studies showed that it was high among psychotic patients as compared to patients with mood disorder [3]. The severity and impact of stigma in patients with a bipolar disorder are more than those in patients with depression [4]. On the other hand, self-stigma was very high as compared to public stigma in patients with depression than in other groups of mental illness and contributing factors included being in the first episode, having a younger age, and having a longer depression episode [5]. However, other findings indicate that no distinction was observed regarding self-stigma between the people with depression and those with bipolar disorders [6].

Societal stigmatizing attitudes towards people with mental illness, such as beliefs that people with mental illness are violent, cannot be treated. The attitude that they are only a burden for the community influences patients' attitudes and beliefs, and they start to internalize and accept how they are labeled because of their illness [7]. Stigma resistance is described as a mentally ill patient's capacity of coping or remaining unaffected and distinguishing and deflecting (“that's not me”) the imposition of different public and self-stigmatizing ideas of their illness [8]. It is an ongoing, active process of using one's experiences, skills, and knowledge to develop a positive identity toward mental illness.

Sex, educational status, social support, employment, self-esteem, current diagnosis, medication adherence, current severity of mental illness, and level of perceived self-stigma were factors associated with stigma resistance in previous studies [6–10]. Studies showed that due to the diminished level of stigma resistance, many domains of a patient's life are disturbed such as their self-esteem, social relationship, work, economy, treatment-seeking behavior, adherence, outcome of treatment, and quality of life [11–12].

Systematic review studies showed that in order to solve this problem, there should be a collaboration between the patient, their family, and different government and nongovernmental institutions to address all forms of stigma imposed on mentally ill patients with the use of the following strategies: intrapersonal interventions such as regularly giving cognitive behavioral therapy and teaching coping mechanisms for addressing internalized and anticipated stigma through challenging stigmatizing thoughts; interpersonal interventions like strengthening social support with their family and intimate friends, forming self-help groups, and communicating through different social media about their illness, personal life, and day-to-day challenges faced; and institutional intervention like giving training to medical staff about the impact of stigma on a patient's life [13–18]. A study done in India showed that conducting an antistigma campaign was taken as an important intervention for changing the attitude and behavior of a community to reduce the stigma of mentally ill patients [12].

Working to enhance the patient's ability to cope against perceived, self, and public stigma of their illness plays a crucial role in fighting stigma and improving their lives as a whole because having high stigma resistance is positively associated with high self-esteem, empowerment, and quality of life [13]. However, stigma resistance has not been explicitly studied except for a few available studies outside Africa. In developing countries, particularly in Ethiopia, there is no previous explicit study reported on stigma resistance among people with a mood disorder. Therefore, the present study is aimed at assessing the stigma resistance among patients with mood disorders and its associated factors.

## 2. Methods and Materials

### 2.1. Study Setting

This facility-based cross-sectional study was conducted at St. Paul's Hospital, Addis Ababa, Ethiopia. It is a referral hospital in the capital city of Ethiopia. The hospital was built in 1969. It has 392 beds, with an annual average of 250,000 patients and a catchment population of over 5 million. It has 13 types of clinical services. Patients with mental illness are treated by psychiatrists and psychiatry residents under the supervision of consultant psychiatrists. The hospital has no inpatient services for mentally ill patients except for those with a problem of substance use disorder for rehabilitation services with 5 beds only. On average, 900 new and old patients with mental illness have been seen per month. Around 11000 new and old patients with mental illness had received treatment per year.

### 2.2. Study Design

An institutional-based cross-sectional study design was employed.

### 2.3. Eligibility Criteria

#### 2.3.1. Inclusion Criteria

All patients with a current diagnosis of bipolar disorder or depression and age above 18 years were included in the study.

#### 2.3.2. Exclusion Criteria

Patients with acute symptoms who need emergency treatment (suicidal patient) were excluded from the study.

### 2.4. Sample Size Calculation

The sample size was calculated by using a single-proportion formula from the findings of a previous study with a proportion of 82.9% [14]:

(1) $$\begin{array}{c} n=\frac{\left(Z\alpha /2\right)2P\left(1-P\right)2}{d2}, \end{array}$$

where *n* = required sample size, *Z* is the reliability coefficient at 95% confidence interval (1.96), and *W* (margin of error) = 0.05.

(2) $$\begin{array}{c} P=0.829=\frac{\left(1.96\right)\left(1.96\right)\left(0.829\right)\left(0.171\right)}{\left(0.05\right)\left(0.05\right)}=217. \end{array}$$

The nonresponse rate was 21 (10%). The total sample size was 217 + 21 = 238 (37% bipolar disorder and 63% depression).

### 2.5. Sampling Procedure

We invited all people with a clinical diagnosis of bipolar disorder or depression aged 18 years and above, who were on follow-up at the psychiatric outpatient department to take part in the study.

We identified all bipolar disorder or depressed patients from their previous chart record. A third year psychiatry resident confirmed their diagnoses using DSM-5 criteria of bipolar disorder and major depression to check its consistency with the earlier diagnosis; if there was a discrepancy between the two diagnoses, we accepted a consultant psychiatrist diagnosis.

### 2.6. Data Collection Instruments

The instruments used for data collection include the following validated tools.

#### 2.6.1. Stigma Resistance Scale

The Stigma Resistance scale is a subcomponent of the internalized Self-Stigma scale which is a 5-item Likert scale that uses the following coding: 1 = strongly disagree, 2 = disagree, 3 = agree, and 4 = strongly agree. Stigma resistance is then summed out of the 20 possible points, and the highest score indicates the highest resistance. The Stigma Resistance subscale is “a person's ability to resist stigma” [15].

#### 2.6.2. Internalized Self-Stigma Scale

The Internalized Self-Stigma scale is a 24-item Likert scale which is used by excluding stigma resistance subcomponents that include the following 4 components: Alienation, Stereotype Endorsement, Perceived Discrimination, and Social Withdrawal. Alienation is “the subjective experience of being less than a full member of society.” Stereotype Endorsement is “the degree to which patients agreed with common stereotypes about people with a mental illness.” Perceived Discrimination experience measures “respondents' perceptions of the way they tend to be treated by others.” Social Withdrawal measures the self-exclusion from social events/situations due to mental illness. Strong internal consistency (*α* = 0.90) and test-retest reliability (*r* = 0.92) have been reported for the ISMI. Each ISMI item contains a declarative statement about a potential stigma issue, and participants respond to each statement by indicating their level of agreement: 1 = strongly disagree; 2 = disagree; 3 = agree; and 4 = strongly agree. The overall Self-Stigma refers to the summed average of the other 4 ISMI subscales excluding the Stigma Resistance subscale (16).

Recent research has suggested that the “Stigma Resistance” subscale is conceptually different from the other subscales [17]. For this reason, stigma resistance (SR) was considered as a separate construct to Self-Stigma throughout this paper [18].

#### 2.6.3. Previous Suicidal Attempt

The question on suicide simply asked whether the participant had felt so desperate that they had attempted suicide (“Have you ever felt so desperate that you even attempted to harm yourself or end your life?”). This question was similar to the single question used in asking about a suicide attempt in the widely used structured Composite International Diagnostic Interview instrument of the World Health Organization [19].

#### 2.6.4. The Rosenberg Self-Esteem Scale

The scale has 10 Likert scale items. Some items are reverse coded. “Strongly Disagree” was given 1 point, “Disagree” was given 2 points, “Agree” was given 3 points, and “Strongly Agree” was given 4 points. The scores for all ten items were summed. Higher scores indicate higher self-esteem [20].

The 12-item interviewer-administered Disability Assessment Schedule version 2, developed by the World Health Organization, was used to establish a level of impairment associated with depression and bipolar disorders. WHODAS assesses the level of disability and the number of days lost from work in the previous 30 days. The instrument is considered cross-culturally applicable [20].

### 2.7. Data Collection Procedures

Face-to-face interviews and document review were used to collect the data for this study. Four BSc nurses and two psychiatry MSc supervisors were recruited, and a two-day training was given about the objective of the study. A self-administered interview was administered and took approximately 30 to 45 minutes to complete; additionally, each data collector at each data collection day reviewed the card and recorded the card number of respondents who had completed the questionnaire and shared the data to all data collectors to avoid redundancy of administering the questionnaire. The principal investigator and the supervisors checked the completeness and quality of the collected questionnaires, and the incomplete questionnaires were removed; feedback was given to data collectors on a daily basis.

### 2.8. Study Variables

#### 2.8.1. Dependent Variable

Stigma resistance was considered a dependent variable in this study.

#### 2.8.2. Independent Variables

Sociodemographic-related factors include age, gender, educational status, marital status, place of residency, and income.

Patient clinical characteristics include current working diagnosis, age at onset of illness, duration of treatment delay, duration of treatment, current clinical status, current functionality, previous suicide attempt, and medication nonadherence status due to stigma.

Psychosocial factors including self-esteem and internalized stigma were the independent variables considered in this study.

### 2.9. Data Analysis

The coded data were entered into EPI-DATA version 3.1 to minimize data entry error and then exported to SPSS version 22.00 for analysis. Descriptive statistics such as texts, percentage, graphs, and tables were used for categorical data, and the mean and standard deviation for continuous data was calculated and used to describe the data. Before performing linear regression analysis, all of the following linear assumptions were met: normality was checked by using the normal histogram curve and Kolmogorov-Smirnov's test; linearity was checked by using a quantile-quantile (QQ) plot and a histogram; the outlier was checked by using an outlier test; multicollinearity was checked using VIF and all variables were VIF < 2; homoscedasticity was checked by using Levene's test wherein all variables were *P* > 0.05, which indicated no heteroscedasticity; and an independent observation was checked by Durbin-Watson's value, and the value of this finding was 1.95. Simple regression was used to identify candidate variables for multiple linear regression at *P* < 0.25, and then to adjust the confounder variables, multiple linear regression analysis was used and variables with*P* < 0.05were assigned to the final model analysis which determined the dependent variable.

### 2.10. Data Quality Assurance

The possible maximum sample size with a 99% nonresponse rate was calculated. Standard and carefully designed questionnaires were used and translated to Amharic by two different persons and back translated to English. The pretest was done among 5% of the participants on Zewditu Hospital who received their treatment at the outpatient service to check for the understandability, reliability, and clarity of the questionnaire. The internal consistency of service satisfaction measurement items in the pretest was Cronbach′s Alpha = 0.824.

The data were collected without wearing a gown outside OPD in the waiting area to prevent reluctance to give reliable information.

### 2.11. Ethical Considerations

Before data collection, ethical clearance was obtained from the Institutional Review Board (IRB) of the Institute of the Health of Addis Ababa University. Permission to conduct the research was obtained from the clinical director of the hospital and the head of the psychiatric clinic. A written consent form was prepared with an outline for the purpose of the study, and this was discussed with each patient who agreed to participate in the study. The patients were assured that they had the right to withdraw from the interview at any time they wish. And they were assured that if they wish to refuse to participate, their care or dignity would not be compromised in any way since there is no relationship between their participation and the health service they received. Patients were informed that there is no expectation of additional treatment or any associated benefits and risks for them when participating in the study. Finally, the questionnaires were locked after data entry was completed.

## 3. Result

### 3.1. Sociodemographic Characteristics of Patients

Out of the 238 samples, 235 patients were enrolled in this study, and the majority of the patients were females (60.4%). The mean age was 37.94 with SD ± 13.2 years and (39.1%) were married (Table 1). Around half of them (42.6%) had no income, and the mean monthly income of the patients was about 59.65$ with (SD ± 85.7) USD.

### 3.2. Clinically Related Factors of the Patients

Nearly two-thirds of the patients (62.6%) had depression, and the mean age of onset of the illness was 27.8 with SD ± 11.2. The mean duration of the illness was 10 with SD ± 9.4 years.

Nearly nine out of ten (86.4%) claimed to be either partially or fully improved. Almost one-third (34: 29.8%) claimed that stigma played a role in their nonadherence (Table 2).

### 3.3. Internalized Stigma Component Result

The overall mean of the internalized stigma (24 items) of mental illness scale was 2.2 with SD = 0.63. Overall, 80.4% of the participants had responded by saying that they agree or strongly agree to at least one item in the Internalized Stigma of Mental Illness Scale (Table 3).

### 3.4. Self-Esteem and Disability

The mean score of self-esteem was 26 with SD ± 4, and the mean percentage score of WHODAS II for the 12 items was 19.4%. The mean of the overall days of disability in 30 days was 10.7 with SD ± 12.05. The mean of the days of total difficulty in 30 days was 1.93 with SD ± 5.26. The mean of the days of partial difficulty in 30 days was 8.17 with SD ± 10.95.

### 3.5. Stigma Resistance

The mean stigma resistance score was 11.9 with SD ± 3, with 49.5% mean percentage score. From all the items used to assess stigma resistance, nearly more than 2/3 of patients responded that they agree and strongly agree to the following items: people with mental illness make important contributions to society, and I can have a good fulfilling life, despite my mental illness (Table 4).

### 3.6. Bivariant Regression Result

Sex, age, job, educational status, monthly income, current psychiatry diagnoses, previous suicidal attempt, duration of illness, duration of treatment, current clinical status, medication nonadherence, self-esteem, disability, and internalized stigma were candidate variables for multiple linear regression at 95% CI, *P* < 0.05.

### 3.7. Multiple Linear Regressions

This study found that having no formal educational status (*B* = −1.465, 95% CI (-2.796, -0.134), *P* ≤ 0.031), WHODAS (II) score (*B* = −0.064, 95% CI (-0.102, -0.026), *P* ≤ 0.001), nonadherence due stigma (*B* = −1.365, 95% CI (-2.151, -0.580), *P* ≤ 0.001), duration of treatment (*B* = 0.091, 95% CI (0.042, 0.14), *P* ≤ 0.001), internalized stigma (*B* = −2.948, 95% CI (-3.642, -2.254), *P* ≤ 0.001), and self-esteem (*B* = 1.859, 95% CI (0.812, 2.906), *P* ≤ 0.001) were significantly associated with stigma resistance score (Table 5).

WHODAS: World Health Organization Disability Rating Scale; 1 = Reff; ^∗∗∗^*P* ≤ 0.001,^∗∗^*P* ≤ 0.01, and ^∗^*P* ≤ 0.05; *α* = constant, step wise analysis; adjusted*R*^2^ = 0.59.3%.

## 4. Discussion

This study is aimed at determining the magnitude and associated factors of stigma resistance among people with a mood disorder at St. Paul's Hospital psychiatry outpatient service.

This study found 49.5% overall percentage score of stigma resistance, and this figure is lower than the study carried out in Europe (59.7%) [21], Singapore (89.2%) [22], and China (76%) [23]. And this variation may be due to the difference in the sociodemographic characteristics of the patients; most of the patients in other study areas have more than a secondary level of education which contributes for better coping with stigma and a good mental health care setting which contributes for better care and treatment outcome.

In this study, patients who had low educational status had less stigma resistance as compared with those who had above the secondary level of education (*B* = −1.465, 95% CI (-2.796, -0.134), *P* ≤ 0.031) as evidenced by the previous study [24], and this may be due to the fact that people who had a higher level of education might have a high level of awareness about their illness and used coping strategies which help to counteract different stereotypes, beliefs, and attitudes coming from the self and public regarding their illness.

This study's findings showed that nearly one third of the patients became medication nonadherent due to stigma, and patients who were nonadherent due to stigma decrease their stigma resistance by 1.36 units (*B* = −1.365, 95% CI (-2.151, -0.580), *P* ≤ 0.01) as compared to those who are not influenced by stigma; this may be because stigma affects a patient's level of self-esteem and self-efficacy and causes demoralization and hopelessness which all leads to medication nonadherence [21, 22].

This study found that when the patients' internalized stigma score increases, their level of stigma resistance decreases by 2.94 units (*B* = −2.948, 95% CI (-3.642, -2.254), *P* ≤ 0.01), and this finding was supported by a previous study [24] and might be explained by the fact that patients' ability of coping towards stigma was influenced by self-attitude and belief of their illness.

Additionally, patients' self-esteem had a positive association with stigma resistance; when patients' self-esteem increases, their stigma resistance level also increases by 1.85 units (*B* = 1.859, 95% CI (0.812, 2.906), *P* ≤ 0.001), and this finding was similar with previous studies done in Israel and Korea [17, 22] and might be explained by the fact that self-esteem is one's sense of power, efficacy, and competency to exert agency, control, or causality within one's environment which affects the person's ability of coping towards different stigma.

Moreover, when patients' duration of treatment increases, their level of stigma resistance increases by 0.09 units (*B* = 0.091, 95% CI (0.042, 0.141), *P* ≤ 0.001) and this might be explained by the fact that the duration of treatment increases their adaptability and acceptance process which help to cope and counteract stigma; so working at an early stage of treatment is very vital in order to enhance stigma resistance as evidenced by the study done in India which found that stigma was high at the early stage of the treatment [5].

Lastly, when patients' disability score increases, their level of stigma resistance decreases by 0.06 units (*B* = −0.064, 95% CI (-0.102, -0.026), *P* ≤ 0.001). This finding is similar with that of the previous study [25] and might explained by the fact that patients' current functionality level has an effect on their stigma resistance level because it affects their self-esteem, self-efficacy, and empowerment; so working on the functionality of patients will help increase their level of stigma resistance.

## 5. Limitation

This study may be affected by recall bias regarding response to the time duration of illness. This study may also have a social desirability bias since the data was collected using face-to-face interviews, and respondents may respond in favor of the interviewer and may either be over- or underreported.

## 6. Conclusion

This study found that half of the participants had stigma resistance. Low educational status, current disability status, duration of treatment, nonadherence due to stigma, and high internalized and low self-esteem were negatively associated with the stigma resistance level among patients with a mood disorder.

Therefore, working on the above-identified factors with different stakeholders will be helpful to enhance the patient's level of stigma resistance which has also a significant impact on patient medication adherence, treatment outcome, and current disability status, especially focusing on the patient's self-esteem and internalized self-stigma which are very vital to enhance patient self-efficacy, empowerment, and self-concept which all increase the patient's stigma resistance level. Moreover, since patient stigma comes from the individual, public, and organizational perspectives, working by involving all of the stakeholders will bring a better outcome. Furthermore, researches with qualitative and quantitative study methods are also suggested to explore the relation of sociodemographic and stigma resistance among patients with mood disorder.

This study result implies that stigma resistance among mentally ill patients is still the problem in our country, and there should be a nationwide program of intervention for enhancing the stigma resistance level of mentally ill patients in the country and worldwide.

## Figures and Tables

**Table 1:** Sociodemographic characteristics of all patients.

Variable	Category	Frequency (*N* = 235)	Percentage (%)
Sex	Male	93	39.6
	Female	142	60.4

Marital status	Single	104	44.3
	Married	92	39.1
	Divorced	19	8.1
	Widowed	20	8.5

Educational status	No education	17	7.2
	Primary	54	23.0
	Secondary	71	30.2
	More than secondary	93	39.6

Occupation	Unemployed	65	27.7
	Housewife	25	10.6
	Student	24	10.2
	Others^∗^	12	4.3
	Government employee	53	22.6
	Private employee	56	23.8

^∗^Others: daily laborer, farmer, retired.

**Table 2:** Clinically related factors of all patients.

Variable	Category	Frequency (*N*=235)	Percentage (%)
Current diagnosis	Bipolar	88	37.4
	Depression	147	62.6

Level of improvement of the patient currently	Fully improved	112	47.7
	Partially improved	91	38.7
	No change	9	3.8
	Relapse	23	9.8

Suicidal attempt	Yes	84	35.7
	No	151	64.3

Medication nonadherence	Yes	114	48.5
	No	121	51.5

Contribution of stigma to nonadherence	Yes	34	29.8
	No	80	70.2

**Table 3:** Response of all patients to each internalized stigma components.

Items of scale	Strongly agree *N* (%)	Agree *N* (%)	Disagree *N* (%)	Strongly disagree *N* (%)
Alienation				
I feel out of place in the world because I have a mental illness	65 (27.7)	80 (34.0)	58 (24.7)	32 (13.6)
Having a mental illness has spoiled my life	34 (14.5)	51 (21.7)	64 (27.2)	86 (36.6)
People without mental illness could not possibly understand me	33 (14.0)	66 (28.1)	71 (30.2)	65 (27.7)
I am embarrassed or ashamed that I have a mental illness	33 (14.0)	92 (39.1)	51 (21.7)	59 (25.1)
I am disappointed in myself for having a mental illness	26 (11.1)	80 (34.0)	64 (27.2)	65 (27.7)
I feel inferior to others who do not have a mental illness	51 (21.7)	100 (42.6)	52 (22.1)	32 (13.6)
Stereotype endorsement				
Stereotypes about the mentally ill apply to me	47 (20.0)	92 (39.1)	48 (20.4)	48 (20.4)
People can tell that I have a mental illness by the way I look	123 (52.3)	77 (32.8)	28 (11.9)	7 (3.0)
Mentally ill people tend to be violent	52 (22.1	83 (35.3)	68 (28.9)	32 (13.6)
Because I have a mental illness, I need others to make most decisions for me	107 (45.5)	110 (46.8)	16 (6.8)	2 (.9)
People with mental illness cannot live a good, rewarding life	79 (33.6)	94 (40.0)	43 (18.3)	19 (8.1)
Mentally ill people should not get married	76 (32.3)	94 (40.0)	40 (17.0)	25 (10.6)
I cannot contribute anything to society because I have a mental illness	70 (29.8)	104 (44.3)	39 (16.6)	22 (9.4)
Discrimination				
People discriminate against me because I have a mental illness	54 (23.0)	106 (45.1)	38 (16.2)	37 (15.7)
Others think that I cannot achieve much in life because I have a mental illness	67 (28.5)	99 (42.1)	47 (20.0)	22 (9.4)
People ignore me or take me less seriously just because I have a mental illness	64 (27.2)	88 (37.4)	52 (22.1)	31 (13.2)
People often patronize me or treat me like a child, just because I have a mental illness	70 (29.8)	96 (40.9)	41 (17.4)	28 (11.9)
Nobody would be interested in getting close to me because I have a mental illness	65 (27.7)	119 (50.6)	38 (16.2)	13 (5.5)
Social withdrawal				
I do not talk about myself much because I do not want to burden others with my mental illness	46 (19.6)	71 (30.2)	74 (31.5)	44 (18.7)
I do not socialize as much as I used to because my mental illness might make me look or behave “weird”	56 (23.8)	109 (46.4)	54 (23.0)	16 (6.8)
I stay away from social situations to protect my family or friends from embarrassment	58 (24.7)	135 (57.4)	33 (14.0)	9 (3.8)
Negative stereotypes about mental illness keep me isolated from the “normal” world	58 (24.7)	126 (53.6)	40 (17.0)	11 (4.7)
Being around people who do not have a mental illness makes me feel out of place or inadequate	58 (24.7)	127 (54.0)	38 (16.2)	12 (5.1)
I avoid getting close to people who do not have a mental illness to avoid rejection	57 (24.3)	125 (53.2)	44 (18.7)	9 (3.8)

**Table 4:** Stigma resistance item response for each patient attending treatment.

Items of scale	Strongly agree *N* (%)	Agree *N* (%)	Disagree *N* (%)	Strongly disagree *N* (%)
I feel comfortable being seen in public with a mentally ill person	9 (3.8)	20 (8.5)	51 (21.7)	155 (66.0)
In general, I can live life the way I want to	35 (14.9)	91 (38.7)	69 (29.4)	40 (17.0)
I can have a good, fulfilling life, despite my mental illness	45 (19.1)	117 (49.8)	44 (18.7)	29 (12.3)
People with mental illness make important contributions to society	47 (20.0)	117 (49.8)	4418.7	27 (11.5)
Living with mental illness has made me a tough survivor	65 (27.7)	43 (18.3)	48 (20.4)	79 (33.6)

**Table 5:** Multiple linear regression analysis.

Model				*B* (95.0% CI)	
		*B*	*P* value	Lower bound	Upper bound
Variables	(Constant)	18.331	0.000^∗∗∗^	16.303	20.359

Educational status	No formal education	-1.465	0.031^∗^	-2.796	-0.134
	Primary	-1.131	0.012^∗^	-2.012	-0.251
	Secondary	-0.565	0.155	-1.345	0.215
	1				

Nonadherence due stigma	Yes	-1.365	0.001	-2.151	-0.580
	1				

WHODAS (II) score		-0.064	0.001^∗∗^	-0.102	-0.026

Self-esteem		1.859	0.001^∗∗^	0.812	2.906

Internalized stigma		-2.948	0.000^∗∗∗^	-3.642	-2.254

Duration of treatment		0.091	0.000^∗∗∗^	0.042	0.141

## Data Availability

The datasets used/or analyzed during the current study are available from the corresponding author on reasonable request.

## Abbreviations

AAU: Addis Ababa University
CI: Confidence interval
ISMI: Self Stigma of Mental Illness Scale
MDD: Major depressive episode
BPD: Bipolar disorder
Mood disorder: Both bipolar disorder and major depressive disorder
SMI: Severe mental illness
SPHMMC: St. Paul's Hospital Millennium Medical College
SR: Stigma resistance
Std. *β*: Standardized *β* coefficients
WHODAS-II: World Health Organization Disability Assessment Schedule, version 2 (the 12 items).
